# A Magnetic Resonance Spectroscopy Study on Polarity Subphenotypes in Bipolar Disorder

**DOI:** 10.3390/diagnostics14111170

**Published:** 2024-05-31

**Authors:** Georgios D. Argyropoulos, Foteini Christidi, Efstratios Karavasilis, Peter Bede, Georgios Velonakis, Anastasia Antoniou, Ioannis Seimenis, Nikolaos Kelekis, Nikolaos Smyrnis, Olympia Papakonstantinou, Efstathios Efstathopoulos, Panagiotis Ferentinos

**Affiliations:** 1 Research Unit of Radiology and Medical Imaging, 2nd Department of Radiology, Attikon General University Hospital, School of Medicine, National and Kapodistrian University of Athens, 115 27 Athens, Greecestratoskaravasilis@yahoo.gr (E.K.); giorvelonakis@gmail.com (G.V.); kelnik@med.uoa.gr (N.K.); sogofianol@gmail.com (O.P.); stathise@med.uoa.gr (E.E.); 2 2nd Department of Psychiatry, School of Medicine, National and Kapodistrian University of Athens, 115 27 Athens, Greece; anastacia345@gmail.com (A.A.); smyrnis@med.uoa.gr (N.S.); pferentinos@med.uoa.gr (P.F.); 3 School of Medicine, Democritus University of Alexandroupolis, 681 00 Alexandroupolis, Greece; 4 Computational Neuroimaging Group, Trinity College Dublin, D08 NHY1 Dublin, Ireland; pbede@tcd.ie; 5 Department of Neurology, St James’s Hospital, D08 W9RT Dublin, Ireland; 6 Medical Physics Laboratory, School of Medicine, National and Kapodistrian University of Athens, 115 27 Athens, Greece; iseimen@med.uoa.gr

**Keywords:** hippocampus, cingulate cortex, emotion regulation network, proton magnetic resonance spectroscopy, bipolar disorder, onset polarity, predominant polarity

## Abstract

Although magnetic resonance spectroscopy (MRS) has provided in vivo measurements of brain chemical profiles in bipolar disorder (BD), there are no data on clinically and therapeutically important onset polarity (OP) and predominant polarity (PP). We conducted a proton MRS study in BD polarity subphenotypes, focusing on emotion regulation brain regions. Forty-one euthymic BD patients stratified according to OP and PP and sixteen healthy controls (HC) were compared. 1H-MRS spectra of the anterior and posterior cingulate cortex (ACC, PCC), left and right hippocampus (LHIPPO, RHIPPO) were acquired at 3.0T to determine metabolite concentrations. We found significant main effects of OP in ACC mI, mI/tNAA, mI/tCr, mI/tCho, PCC tCho, and RHIPPO tNAA/tCho and tCho/tCr. Although PP had no significant main effects, several medium and large effect sizes emerged. Compared to HC, manic subphenotypes (i.e., manic-OP, manic-PP) showed greater differences in RHIPPO and PCC, whereas depressive suphenotypes (i.e., depressive-OP, depressive-PP) in ACC. Effect sizes were consistent between OP and PP as high intraclass correlation coefficients (ICC) were confirmed. Our findings support the utility of MRS in the study of the neurobiological underpinnings of OP and PP, highlighting that the regional specificity of metabolite changes within the emotion regulation network consistently marks both polarity subphenotypes.

## 1. Introduction

Over the past decade, magnetic resonance imaging (MRI) has highlighted structural and functional changes in neuropsychiatric diseases, making MRI a strong candidate as a technique that can provide potential biomarkers and contribute to our understanding of the neuropathophysiological processes in neuropsychiatric diseases [1,2,3]. Proton magnetic resonance spectroscopy (MRS) is a non-invasive technique to determine in vivo the chemical composition, the metabolic function, the neurotransmitter level, and the neural integrity of the tissue based on the assessment of the metabolite concentration in the tissue of interest [4,5,6]. The most commonly measured metabolites are N-acetyl-aspartic acid (NAA) (an indicator of neural integrity of the brain), creatine (Cr) (an indicator of phosphate metabolism), choline (Cho) (an indicator of membrane breakdown and cell death), myo-Inositol (mI) (an osmolyte and a marker of astrocytic activity), and the neurotransmitters gamma-aminobutyric acid (GABA), glutamine (Glu), and glutamate (Gln) [7].

Affective disorders are one of the leading causes of morbidity and mortality after cardiovascular diseases, cancer, and road traffic accidents. Bipolar disorder (BD) belongs to affective disorders and is characterized by emotional dysregulation [8,9,10], affecting roughly >1% of the global population, regardless of sex, ethnicity, or social income [11,12,13,14]. It is associated with significant psychosocial morbidity and mortality due to increased suicidality and medical poly-comorbidity [12,13,14]. Up to 50% of BD patients have visited at least three clinicians prior to diagnosis and 10% of BD patients report at least 10 visits prior to a proper diagnosis [15,16]. Therefore, there is a significant delay between the onset of first symptoms and accurate/definitive diagnosis ranging between 5 and 10 years [15,16,17]. Delayed diagnosis of BD is often associated with prolonged periods of destabilization, residual symptoms, increased psychosocial morbidity [18], and development of treatment resistance [19,20]. In addition, it is associated with increased treatment costs [18] in contrast to early and accurate recognition of symptoms and diagnosis of BD, which significantly reduce these costs.

In the context of the clinical and neurobiological definition of BD, the conceptual construct of “predominant polarity” (PP) has been considerably used in recent years [21]. PP is defined as polarity that occurs during at least two-thirds of lifetime mood episodes and distinguishes patients with BD into three PP subgroups: patients who experience predominantly depressive episodes (PP-D), patients who experience predominantly manic or hypomanic episodes (PP-M), and patients who do not meet any of the aforementioned criteria and present an unspecified polarity (PP-U) [21]. PP has a high clinical importance regarding disease course management [22,23,24,25], having an important role in BD maintenance therapy through the recently proposed “polarity index” (PI) [22]. Equally important for disease course, PP, and overall prognosis [26,27] is also considered to be the “onset polarity” (OP) [28,29]. The latter is defined as the polarity of the first episode in BD, thus leading into two OP subgroups, i.e., depressive onset polarity (OP-D) and manic onset polarity (OP-M).

Recent MRS studies have offered the potential for in vivo measurements of brain chemical profile in BD and have enhanced our understanding of BD neuropathophysiological mechanisms [30,31]. Reduced NAA is reported in the hippocampus, frontal, and occipital regions [31,32,33,34,35], suggesting neuronal or axonal loss or mitochondrial dysfunction [36]. Several studies also show reduced levels of Cr in the lateral prefrontal cortex, hippocampus, and basal ganglia. There are inconsistent findings so far regarding Cho levels in the lateral prefrontal cortex, hippocampus, and anterior cingulate cortex [37,38,39,40,41]. On the other hand, an increase in Cho levels in basal ganglia has been reported [40]. A previous review highlighted abnormal mI concentrations in manic or depressed BD patients, mainly in frontal and temporal lobes, cingulate gyrus, and basal ganglia, with these abnormalities disappearing in euthymic patients, possibly due to a normalizing effect of the treatment [42]. Increased Glu and Gln levels are reported in the cingulum, in the prefrontal, parietal, occipital, and hippocampal regions [37,43,44,45].

The neurobiological underpinnings of PP and OP have only recently been attempted to be determined in neuroimaging studies, which to date have focused on structural changes (gray matter density or cortical thickness) in the hippocampus [46], cerebellum [47], and cerebral hemispheres [48], as well as on white matter integrity in efferent and afferent cortico-cerebellar tracts [47] and major commissural, associative, and projection tracts [48]. Not only common but also distinct patterns of neuroanatomical changes have been reported between the PP [46,47,48] and the OP subphenotypes [47,48]. However, there are no data to date on the profile of metabolites in relation to OP and PP, especially in regions involved in the pathophysiology of BD, such as the hippocampus and the cingulate cortex [49].

Much brain MRS research in BD covers a small number of metabolites examined in a few brain regions, often one. Reports on the effect size of differences between polarity subphenotypes over a range of metabolites may be useful for researchers and clinicians, allowing a better understanding of the neuroimaging underpinnings of polarity subphenotypes. The main objective of the present study is to investigate the profile of major metabolites in euthymic BD patients in association with polarity subphenotypes (OP and PP), using proton MRS and focusing on brain regions that are implicated in the pathophysiology of the BD, i.e., hippocampus and cingulate cortex.

## 2. Materials and Methods

### 2.1. Ethics Approval

The study has been approved by the institutional review board of Attikon General University Hospital (ΨΥΧ, ΕΒΔ654/01-10-20218) and was conducted in accordance with the Declaration of Helsinki. All participants provided informed consent before inclusion.

### 2.2. Participants

Forty-one euthymic BD patients were included in this single-center neuroimaging study. All patients were recruited from the 2nd Department of Psychiatry, NKUA (Attikon General University Hospital) during a period of 12 months. All patients were diagnosed with either type I BD (BD-I, *n* = 30) or type II BD (BD-II, *n* = 11) according to DSM-5 criteria [8], and they all attended a specialized outpatient clinic. Sixteen age- and gender-matched healthy controls (HCs), who were unrelated to the patients, were also recruited for the purpose of the MRS analysis. Inclusion criteria for all participants were age ≥ 18 years, education > 3 years, Greek as a native language, and right-handedness. Exclusion criteria for BD patients were serious neurologic or neurodevelopmental disorders (e.g., autism) and a history of substance/alcohol misuse during a period of 6 months preceding recruitment. Exclusion criteria for HC were any neurologic and psychiatric diagnosis, history of substance/alcohol abuse, major untreated organic disorders, developmental abnormalities, and family history of major psychiatric disorders in first-degree relatives [50].

### 2.3. Clinical Evaluation

Each patient underwent a standardized clinical examination, as previously described in detail [51]. BD patients’ lifetime and current diagnosis status were verified using SCID-5 [52]. According to the DSM-5, none of the patients suffered a serious neurocognitive decline [8]. Evaluation of current clinical status was conducted within ± 3 days of the MRI examination. This involved administering the 17-item Hamilton Depression Rating Scale (HDRS; cut-off ≤ 7) [53] and the Young Mania Rating Scale (YMRS, cut-off ≤ 12) [54]. For all patients, the following clinical characteristics were recorded in detail: disease duration, the number of previous episodes of depression, as well as mania and hypomania (i.e., hyperthymic episodes), OP, PP, the number of hospitalizations, the lifetime occurrence of psychotic symptoms, lifetime Axis I comorbidities, current medication (lithium, anticonvulsants, antidepressants, antipsychotics), and family history of psychiatric disorders in first-degree relatives (BD, schizophrenia, major depressive disorder) [50]. All BD patients were classified as PP-D or PP-M if at least two-thirds of all their episodes were of the same polarity (i.e., depressive or manic, respectively). Patients with unspecified PP (PP-U) were defined as BD patients who did not fit the requirements for PP-D or PP-M. Based on the polarity of the first episode (i.e., OP), all patients were also classified as either manic (OP-M) or depressed (OP-D) OP. HC was evaluated by a brief clinical interview based on SCID-5 [52].

### 2.4. MRI Data Acquisition

**Figure 1:**
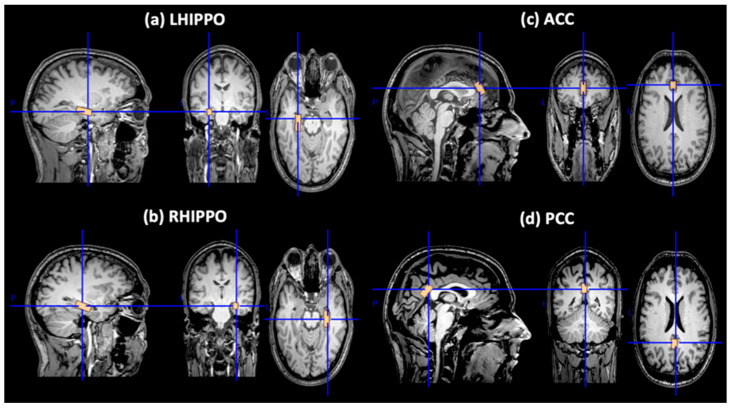
1H-MRS voxel position in (**a**) LHIPPO, (**b**) RHIPPO, (**c**) ACC, and (**d**) PCC (data from a healthy control). 1H-MRS = proton magnetic resonance spectroscopy; LHIPPO = left hippocampus; RHIPPO = right hippocampus; ACC = anterior cingulate cortex; PCC = posterior cingulate cortex. The voxel size for each brain region in Figure 1 was selected for visualization purposes and does not correspond to the voxel size during the MRS acquisition, which is presented in the text.

All participants underwent the same standardized whole-brain imaging protocol on a 3 T Philips Achieva-Tx MR scanner (Philips, Best, The Netherlands) equipped with an eight-channel head coil. For each participant, the head was positioned in the scanner by placing foam wedges on both head sides to immobilize the head in the coil. We applied a 3D high-resolution T1 (3D-HR-T1) weighted sequence (inversion time: 1200 ms, repetition time (TR): 9.9 ms, echo time (TE): 3.7 ms, flip angle: 7°, voxel-size: 1 × 1 × 1 mm, matrix size: 244 × 240, 170 slices), and a T2-weighted fluid-attenuated inversion recovery (T2-FLAIR) sequence (TR: 11,000 ms, TI: 2800 ms, TE: 125 ms, acquisition matrix: 384 × 186, slice thickness 4 mm). T2-FLAIR was used to exclude severe cerebrovascular or incidental neuroinflammatory disease according to standard clinical neuroradiological criteria on visual inspection by two experienced radiologists (O.P. and G.V.). Single-voxel point resolved spectroscopy (PRESS) pulse sequence was used for spectrum acquisition with TR = 2000 ms, TE = 35 ms, and NSA = 256 combined with water suppression chemically selective saturation pulses to suppress the water signal (Philips EXCITATION method). During the acquisition preparation phase, the full amplitude of the water curve at half maximum (FWHM) on the MRI screen served as the initial quality indicator for evaluating the local homogeneity of the field. A cut-off value of 15 Hz was used. For the purpose of the present study, we used the following MRS voxels: left hippocampus (LHIPPO): 9 mm (RL) × 23 mm (AP) × 8 mm (FH), right hippocampus (RHIPPO): 9 mm (RL) × 23 mm (AP) × 8 mm (FH), anterior cingulate cortex (ACC): 10 mm (RL) × 10 mm (AP) × 20 mm (FH), and posterior cingulate cortex (PCC): 10 mm (RL) × 10 mm (AP) × 20 mm (FH) (Figure 1). The total acquisition time for the MRS protocol was 44 min, including 5 startup acquisitions for each voxel and chemically selective saturation pulses. A healthy participant was scanned three times with a two-week interval between each examination to address issues related to sequence optimization and consistency of voxel placement (repeatability).

### 2.5. MRS Data Analysis

**Figure 2:**
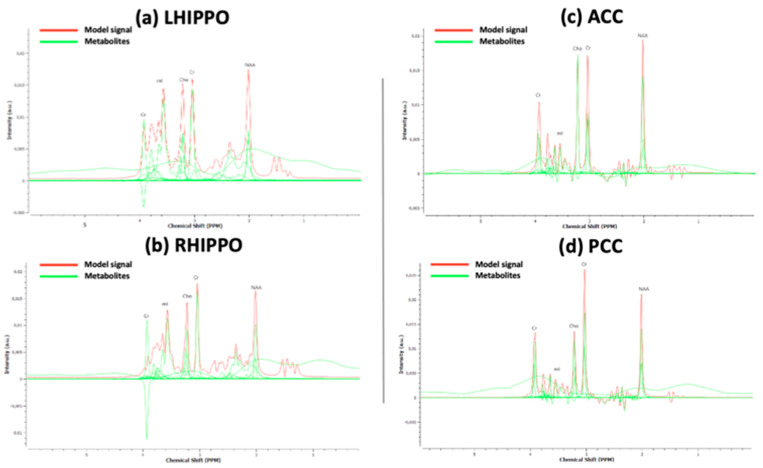
A representative spectrum with the fitted peaks (Tarquin software) for (**a**) LHIPPO, (**b**) RHIPPO, (**c**) ACC, and (**d**) PCC. LHIPPO = left hippocampus; RHIPPO = right hippocampus; ACC = anterior cingulate cortex; PCC = posterior cingulate cortex.

Raw spectroscopy data were extracted from the MRI scanner, and the metabolite concentrations (mM) were quantified using TARQUIN (version 4.3.10) [55]. Based on widely adopted spectral quality criteria [56], we excluded four participants (three BD patients and one HC) from further analyses. These exclusion criteria are based on calculated TARQUIN quality parameters FWHM < 0.15 ppm, SNR > 5, and measure of fit quality <2.5 for quantification reliability and spectral quality [56]. Based on the only available option in Tarquin software version 4.3.10, the voxel water signal was used as a reference signal for estimating metabolite concentration. To our knowledge, most research groups use water as a reference metabolite when estimating absolute metabolite concentrations [57,58]. During the pre-processing steps, the spectroscopic data were corrected for eddy currents and frequency drifting with 1H NAA Cr Cho internal base as the reference signal. Accurate baseline modeling is crucial, especially at short TE, to proceed with reliable spectroscopy analysis [55]. Considering that we regarded lipids as metabolites of no particular interest, the lipid filter was chosen active and the internal base was set as 1H brain + Glutathione (Glth) + no Lip/MM to reduce the risk of modeling noise (baseline overfitting) [55]. All other parameters remained the same as the default. In the calculation of absolute concentration values, we applied the correction factor as previously used [58] to account for the different distribution of metabolites in CSF, GM, and WM tissues, including the MRS voxel. The CSF, GM, and WM fractions were calculated using a previously published Matlab code, which was developed by Dr. Nia Goulden and Dr. Paul Mullins at Bangor University (UK) [59,60]. A representative spectrum with the fitted peaks (Tarquin software) for each region is provided in Figure 2. Concentrations of metabolites were exported and are expressed in units of mM. The following metabolites were included in further analyses: total NAA (tNAA): N-acetylaspartate (NAA) + N-acetylaspartateglutame (NAAG), total Cho (tCho): glycerophosphocholine (GPC) + phosphocholine (PCH), total Cr (tCr): creatine (CR) + Phospho-creatine (PCR), and mI. We also calculated the following ratios using the absolute values of the metabolites: tNAA/tCho, tNAA/tCr, tCho/tCr, mI/tNAA, mI/tCho, and mI/tCr.

### 2.6. Statistical Analysis

Normality assumptions were tested for the dependent variables (metabolites), and then, further parametric statistical criteria were applied. Differences in age, education, and gender distribution between HC and polarity subgroups were examined by one-way analysis of variance (ANOVA; age, education) and χ^2^-test (sex). To test the effect of polarity subphenotypes (OP and PP) on the metabolites of HIPPO bilaterally (i.e., LHIPPO, RHIPPO), ACC, and PCC, we performed a series of multivariate analyses of covariance (MANCOVA) separately for the metabolite absolute values and the ratios using them as dependent variables, the grouping variable (OP (HC, OP-D, OP-M) or PP (HC, PP-D, PP-M, PP-U)) as independent variable, and age, sex, and education as covariates. In case of a significant MANCOVA Pillai’s Trace, metabolites (absolute values or ratios) with a significant univariate omnibus test (main effect) were identified, and post hoc comparisons between subgroups were performed applying Bonferroni correction for multiple tests to reduce type I error. Furthermore, Cohen’s d effect sizes were calculated for pairwise comparisons between subgroups for any metabolite found to be significantly affected by either OP or PP. A |d| value of 0.80 or higher is considered a large effect size, a |d| value between 0.50 and 0.79 is considered a medium effect size, a |d| value between 0.20 and 0.49 is considered a small effect size while a |d| value ≤ 0.19 is considered a negligible effect size. As a follow-up analysis, effect sizes in OP post hoc pairwise comparisons were compared to effect sizes in PP pairwise comparisons, and consistency of agreement was calculated with a two-way mixed effects ICC [61,62]. An ICC < 0.40 indicates poor reliability, an ICC between 0.40 and 0.59 indicates fair reliability, an ICC between 0.60 and 0.74 indicates good reliability, and an ICC > 0.75 indicates excellent reliability [63]. All analyses were performed using IBM SPSS v. 28.

## 3. Results

### 3.1. Demographic and Clinical Characteristics

The demographic characteristics of patients with BD and HC, as well as the basic clinical characteristics of the OP/PP polarity subgroups, are presented in Table 1. With regard to OP (OP-M, *n* = 17; OP-D, *n* = 24), no significant differences were found in age, education, and gender distribution. With regard to PP (PP-M, *n* = 12; PP-D, *n* = 14; PP-U, *n* = 15), a significant difference in age was found only between patients with PP-M and patients with PP-D (*p* = 0.009; PP-D > PP-M). Comparisons in education and gender distribution were not significant. We did not find significant between-group differences in disease duration regarding OP and PP. Further analyses between the subphenotypes of OP and PP on individual clinical variables revealed significant differences in the BD subtype (*p* < 0.001 for OP, *p* = 0.004 for PP), the number of depressive episodes (PP, *p* < 0.001), the number of hyperthymic episodes (PP, *p* = 0.013), and history of suicide attempts (OP, *p* = 0.001).

We also examined a crosstabulation of OP and PP subphenotypes (Table 2). OP and PP subgroups were significantly correlated (Fisher’s exact *p* = 0.007). Most (>50%) OP-M subjects ended up as PP-M and most (50%) OP-D subjects as PP-D while around 35% of both OP-M/OP-D subjects ended up as PP-U and even less (around 12%) converted to opposite polarity PP (OP-M to PP-D and OP-D to PP-M). The PP-M subgroup consisted mainly (75%) of OP-M subjects and the PP-D subgroup mainly (85.7%) of OP-D subjects. PP-U included slightly more (60%) OP-M subjects.

### 3.2. MRS in Cingulum (ACC, PCC) and Bilateral Hippocampus

#### 3.2.1. Reproducibility Study of MRS Voxel Placement

Qualitative assessment of the voxel placement by two independent neuroradiologists (G.V., O.P.) provided evidence of the consistency of voxel placement. Furthermore, ICC values for each metabolite for the MRS voxels were >0.95, indicating excellent consistency.

#### 3.2.2. Onset Polarity (OP)

Table 3 presents the profile of differences in ACC, PCC, LHIPPO, and RHIPPO in OP subgroups and HC.

We found a significant main effect of OP on the metabolite profile based on absolute values (Pillai’s Trace = 0.881, F = 1.672, *p* = 0.039, partial η^2^ = 0.440). In particular, we detected significant differences in ACC mI (*p* = 0.038) and PCC tCho (*p* = 0.033) and a trend towards significance in PCC tCr (*p* = 0.081), LHIPPO mI (*p* = 0.061), and RHIPPO tCr (*p* = 0.054). Based on post hoc comparisons with Bonferroni correction, we observed significant differences in PCC tCho (HC < OP-M, *p* = 0.043). Marginal differences were found in ACC mI (OP-M > OP-D, *p* = 0.056), PCC tCr (HC < OP-M, *p* = 0.078), LHIPPO mI (OP-M < OP-D, *p* = 0.072), and RHIPPO tCr (HC > OP-M, *p* = 0.056).

We also found a significant main effect of OP on the metabolite profile based on ratios (Pillai’s Trace = 1.330, F = 2.148, *p* = 0.004, partial η^2^ = 0.665). In particular, we detected significant differences in ACC mI/tNAA (*p* = 0.003), ACC mI/tCr (*p* = 0.005), and ACC mI/tCho (*p* = 0.004), as well as RHIPPO tNAA/tCho (*p* = 0.035) and RHIPPO tCho/tCr (*p* = 0.005). Based on post hoc comparisons with Bonferroni correction, we observed significant differences in ACC mI/tNAA (OP-M > OP-D, *p* = 0.002), ACC mI/tCr (OP-M > OP-D, *p* = 0.005), ACC mI/tCho (HC > OP-D, *p* = 0.016), ACC mI/tCho (OP-M > OP-D, *p* = 0.010), RHIPPO tNAA/tCho (HC > OP-M, *p* = 0.038), and RHIPPO tCho/tCr (HC < OP-M, *p* = 0.008; OP-M > OP-D, *p* = 0.024). A marginal difference was found in ACC mI/tCr (HC > OP-D, *p* = 0.076).

#### 3.2.3. Predominant Polarity (PP)

Table 4 presents the profile of differences in ACC, PCC, LHIPPO, and RHIPPO in PP sugroups and HC.

We did not find a significant effect of PP on the metabolite profile neither using the absolute values (tNAA, tCho, tCr, mI), Pillai’s Trace = 0.993, F = 1.052, *p* = 0.408, partial η^2^ = 0.331, nor the ratios (tNAA/tCho, tNAA/tCr, tCho/tCr, mI/tNAA, mI/tCho, mI/tCr), Pillai’s Trace = 1.588, F = 1.218, *p* = 0.196, partial η^2^ = 0.529.

#### 3.2.4. Follow-Up Analysis Based on Effect Sizes

Effect sizes for pairwise comparisons were calculated for all metabolites (absolute values and/or ratios) for which significant or marginally significant main effects of OP were identified. As a follow-up analysis, effect sizes were also calculated for the same metabolites (absolute values and/or ratios) in PP pairwise between-group comparisons (Table 5).

Large effect sizes (|d| ≥ 0.80, brown color—Table 4) were found in the following comparisons:

- HC vs. OP-M: PCC tCho (d = −0.89), and RHIPPO tCr (d = 0.85), tNAA/tCho (d = 0.90), and tCho/tCr (d = −1.00).
- HC vs. OP-D: ACC mI/tCr (d = −1.00)
- OP-M vs. OP-D: ACC mI (d = 0.81), mI/tNAA (d = 1.12), mI/tCho (d = 1.04), and mI/tCr (d = 1.00), RHIPPO tCho/tCr (d = 0.91)
- HC vs. PP-M: RHIPPO tCr (d = 1.05) and tCho/tCr (d = −0.86)
- HC vs. PP-D: ACC mI/tCr (d = 0.86)
- HC vs. PP-U: PCC tCr (d = −0.80) and tCho (d = −0.88)
- PP-M vs. PP-D: RHIPPO tCr (d = −0.99)

Medium effect sizes (0.79 ≤ |d| ≥ 0.50, orange color—Table 4) were found in the following comparisons:

- HC vs. OP-M: ACC mI/tNAA (d = −0.53), PCC tCr (d = −0.79), and LHIPPO (d = 0.61)
- HC vs. OP-D: ACC mI (d = 0.72), mI/tNAA (d = 0.63), mI/tCho (d = 0.77)
- OP-M vs. OP-D: PCC tCho (d = 0.68), LHIPPO mI (d = −0.77), RHIPPO tCr (d = −0.54) and tNAA/tCho (d = −0.61)
- HC vs. PP-M: RHIPPO tNAA/tCho (d = 0.63)
- HC vs. PP-D: ACC mI (d = 0.62) and mI/tCho (d = 0.64), PCC tCr (d = −0.63), and RHIPPO tNAA/tCho (d = 0.65)
- PP-M vs. PP-D: ACC mI/tNAA (d = 0.76) and mI/tCho (d = 0.59)
- PP-M vs. PP-U: ACC mI/tNAA (d = 0.54), and RHIPPO tCr (d = −0.56)
- PP-D vs. PP-U: ACC mI (d = −0.59), mI/tCho (d = −0.66), and mI/tCr (d = −0.76), and PCC tCho (d = −0.61).

All other comparisons yielded small (0.49 ≥ |d| ≥ 0.20) or negligible (|d| ≤ 0.19) effect sizes.

**Figure 3:**
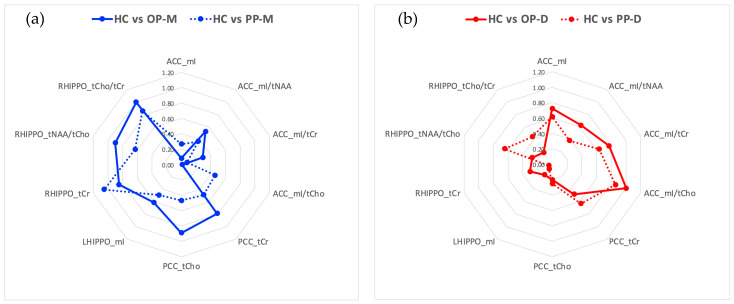
The pattern of the magnitude of absolute differences (absolute Cohen’s |d| effect size) (**a**) between HC and manic subgroups (OP-M and PP-M) and (**b**) between HC and depressive subgroups (OP-D and PP-D).

By examining the pattern of the magnitude of differences (absolute Cohen’s |d| effect size) (Figure 3), we observed that compared to HC the manic subphenotypes (i.e., OP-M and PP-M) showed greater differences in hippocampal regions (right hemisphere) and PCC (though the largest appeared in the RHIPPO), whereas the depressive suphenotypes (i.e., OP-D and PP-D) showed greater differences in ACC. PP-U showed an intermediate pattern of changes between the PP-M and the PP-D, with greater differences in PCC, as shown in Table 4.

**Figure 4:**
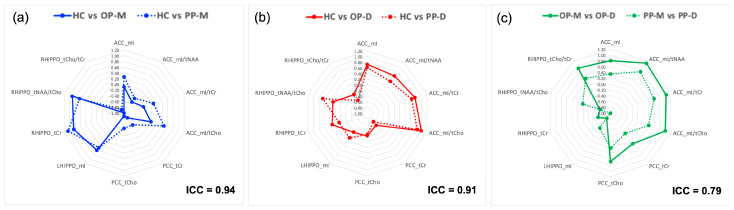
The pattern of the magnitude of differences (Cohen’s d effect size) followed up from OP to PP between-group comparisons (**a**) between HC and manic subgroups (OP-M and PP-M), (**b**) between HC and depressive subgroups (OP-D and PP-D), and (**c**) between manic and depressive OP and PP subgroups.

As shown in Table 2, most OP-M/OP-D subjects preserved their polarity in PP. Therefore, we finally assessed the consistency of agreement (ICC) between the effect sizes in OP and PP pairwise comparisons, focusing on comparisons involving the same polarities (i.e., OP-M and PP-M vs. HC, OP-D and PP-D vs. HC, OP-M vs. OP-D, and PP-M vs. PP-D). Our analysis showed that the pattern of the magnitude of differences between HC and OP-M as well as between HC and OP-D is reliably replicated when HC and PP-M (ICC = 0.94; excellent) and HC and PP-D (ICC = 0.91; excellent) are compared, respectively. In addition, the pattern of magnitude of differences between OP-M and OP-D is reliably replicated when PP-M and PP-D are compared (ICC = 0.79; excellent). Although effect sizes are consistent between OP and PP, they are reduced in PP compared to OP. Figure 4 shows the above-mentioned concordances.

## 4. Discussion

In the present study, we examined the metabolite pattern with regard to BD polarity subphenotypes (PP and OP) focusing on brain regions that are traditionally implicated in BD as part of the emotion regulation network, namely the cingulate gyrus (ACC and PCC) and hippocampus (LHIPPO and RHIPPO). By assessing a set of metabolites in a variety of brain regions rather than focusing on a single region of interest, we were able to investigate distributed patterns of metabolite–polarity associations. Based on the main analysis of the absolute values and ratios, our study suggests that ACC mI, mI/tNAA, mI/tCho, and mI/tCr, PCC tCr and tCho, LHIPPO mI, and RHIPPO tCr, tNAA/tCho and tCho/tCr may differentiate polarity subphenotypes in BD. The effect sizes and our follow-up reliability analysis further provide an easy-to-understand lookup for researchers and clinicians, highlighting that (1) there may be a regional specificity of manic and depressive polarity (compared to HC) within the emotion regulation network, with manic polarity mostly linked to hippocampal and PCC changes and depressive polarity mostly linked to ACC changes, and (2) the magnitude of several metabolite differences consistently marks both polarity subphenotypes. The present results highlight the importance of studying both OP and PP in BD, expanding the existing field of neuroimaging studies on the neurobiological substrate of BD polarity [46,47,48] and providing further evidence on the emotion dysregulation network in BD [64].

### 4.1. Metabolite Changes in Cingulate Cortex (ACC, PCC) and Hippocampus (HIPPO R and L)

The cingulate cortex, mainly divided into an anterior (ACC) and a posterior (PCC) region, subserves cognitive and affective processing [65,66]. ACC has extensive connections with areas known to be important for emotion (e.g., amygdala), autonomic (e.g., lateral hypothalamus, brainstem centers), memory (e.g., hippocampal region), and reward (e.g., orbitofrontal cortex, ventral striatum) related functions. The dorsal ACC which broadly corresponds to the MRS voxel of our study is part of the default-mode network (DMN). PCC is another hub center of the DMN and constitutes an important efferent pathway to the HIPPO. PCC represents one of the most metabolically active brain areas at rest and is highly associated with several cognitive processes, including attention, episodic memory, self-monitoring and self-awareness, regulation of emotion, action, and cognition [66]. In the framework of emotional processing, PCC is implicated in the assessment of the self-relevance of emotional stimuli and events [67]. On the other hand, the HIPPO has been implicated not only in memory [68,69,70] but also in mood processing and especially, the regulation of affective states and emotional behavior that facilitates emotionally appropriate behavior in certain contexts [71,72]. To date, both animal and human studies support the importance of hippocampal–cingulate networks for memory and emotion [73].

We demonstrated significant differences and/or medium-to-large effect sizes in mI, mI/tNAA, mI/tCho, and mI/tCr ratios mostly in ACC and to a lesser degree in LHIPPO. These differences were more profound in OP (*p*-values, effect sizes) and to a lesser degree in PP (effect sizes). The mI has been proposed as a glial cell marker [74], exerts an osmolyte role, and functions as a form of glucose storage [75]. It is also considered as a precursor of phosphatidylinositol, which is a component of phospholipid membranes, and as a substrate for the secondary phosphoinositide transporters [76,77]. Changes in mI levels may be associated with abnormal phospholipid metabolism and intracellular signal transmission systems. In fact, preclinical evidence in BD suggests that mI depletion may be the underlying mechanism through which lithium acts in patients with BD [78,79]. Alterations in mI are rarely reported in euthymic BD patients, but it appears that the concentration is decreased in depressed BD and increased in manic BD compared to healthy individuals [42]. Thus, the pattern of our findings is in line with previous studies, since we mostly found increased concentration in OP-M compared to HC and OP-D, mainly in ACC. Of note, future studies may also examine the phospholipid membrane metabolism in association with polarity subphenotypes using phosphorus MRS [31P-MRS], since data related to OP and PP are lacking.

We observed increased tCr in PCC and decreased tCr in RHIPPO mostly in OP-M compared to HC and to a lesser degree in OP-D compared to HC. Cr indicates phosphate metabolism, and it is important in the storage and transfer of energy. The 1H-MRS signal related to phosphocreatine (PCr) and Cr (tCr) is generally considered a measure of overall brain health, and reduced tCr often indicates impaired function or integrity. In many major psychiatric disorders, tCr alterations are regional and usually state-dependent [80]. Altered brain Cr cycle metabolites have generally been reported in BD, but specific data in PCC and HIPPO are limited or lacking. Decreased peak PCr [81,82,83,84] or tCr (Cr and PCr) [85,86,87] in the frontal lobe has been reported in BD while findings in ACC are still inconclusive [88,89]. Of note, in a recent study on ACC, decreased Cr and tCr were found in euthymic BD compared to healthy individuals, while increased PCr and lower Cr/PCr ratio in ACC were associated with greater severity of physical and sexual abuse in the BD group only [90]. With regard to the PCC, an area with increased tCr in our study in both manic and depressive polarities, few studies are available in BD while data on the metabolite profile of PCC are limited [91,92,93]. Previous studies focusing on PCC in BD and unipolar depression groups [92,93] or acutely manic BD and acutely ill patients with schizophrenia [89] did not find Cr changes in BD. However, abnormal functional alterations in PCC have been reported in BD. For example, a study in euthymic BD patients did not identify changes in the medial frontal or lateral parietal nodes of the DMN during a task fMRI but identified failure of de-activation in the retrosplenial cortex and adjacent precuneate cortex, close to the posterior midline node of the PCC [94]. A previous fMRI study found greater de-activation in patients with first-episode mania compared to healthy individuals in bilateral PCC [95]. A recent resting-state fMRI study in first-episode, drug-naïve manic BD patients found increased neural activity (Regional Homogeneity) in PCC, which could effectively discriminate BD and HC with >80% accuracy, sensitivity, and specificity [96]. We also found decreased tCr in RHIPPO in manic polarity (OP-M and PP-M) compared to HC (large effect sizes). Limited data are available regarding tCr alterations in HIPPO in patients with BD. In a previous study in BD-I patients, Haarman and colleagues also found decreased tCr and tNAA in BD compared to healthy individuals in LHIPPO [97].

Neurons themselves cannot synthesize Cr, which is transferred from blood plasma by specific creatine transporters (CRTs) and enters the brain via these specialized CRTs at the blood–brain barrier. After crossing the blood–brain barrier, Cr is taken up from extracellular fluid by neurons and oligodendrocytes by CRTs, and it fulfills its fundamental function as an energy shuttle and homeostasis regulator. The highest levels of CRT expression are found within the olfactory bulb, the granulate cells of the hippocampal dentate gyrus, the pyramidal cells of the cerebral cortex, the cerebellar Purkinje cells, the motor and sensory cranial nerves of the brain stem, and the dorsal and ventral horns of the spinal cord, while the lowest levels of CRTs concentration are found in the basal ganglia and white matter [98]. Pyramidal cell structure differs between ACC and PCC, two regions involved in the pathophysiology of BD [99]. ACC is characterized by a lower cell number per unit volume but a higher complexity of the dendritic arborization, while PCC is characterized by a higher cell packing density but a lower degree of dendritic arborization [100,101]. These structural differences, which are also associated with functional differences between the two parts of the cingulate cortex, may further explain the regional specificity of Cr, PCr, or tCr changes in BD studies.

We also identified significant differences and/or medium-to-large effect sizes in tCho and tCho/tCr in PCC and RHIPPO, respectively. When present, changes in Cho are thought to primarily reflect changes in phospholipid membrane metabolism [102]. Several studies have generally reported increased Cho concentration in patients with BD [40,103,104]. There are also reports of increased Cho concentration in the hippocampus, even in euthymic patients with BD [45] or patients with bipolar depression [105]. Given that Cho is considered a marker of phospholipid membrane metabolism, the observed elevated levels in our study probably indicate increased membrane breakdown in PCC and hippocampus in manic and to a lesser degree depressive polarities compared to healthy individuals. In a recent study on BD and unipolar depression, Kong and colleagues reported increased Cho in PCC in the BD group but not in unipolar depression compared to healthy individuals [91].

From a methodological point of view, it is important to note that Cr is predominantly used as an internal standard, allowing for ratio calculations (i.e., metabolites/Cr ratios) based on the assumption that it tends to be maintained at a relatively constant level under various conditions. In addition, Cho is also used as a denominator for other metabolites in spectroscopic studies, assuming that it does not change. However, spectroscopic studies both in neurological and psychiatric diseases highlight changes in Cr and Cho, thus calling into question their use as an internal standard for calculating ratios to represent absolute metabolite changes. Our study further supports that absolute values should separately be examined because Cr and Cho levels may not remain stable and may not show a uniform pattern of change across different regions.

We also found significantly decreased tNAA/tCho in RHIPPO in OP-M compared to HC (*p*-values, large effect size). Reduced NAA/Cr but not NAA/Cho has been found in bilateral hippocampi in patients with BD-I compared to healthy individuals [33]. Most studies to date generally report reduced NAA concentration in BD, mainly in frontal regions and the HIPPO, although there are also studies that do not report changes in NAA [106,107]. Of note, NAA in the HIPPO was found to be reduced as a function of increased mania in a recent study [108], whereas reduced NAA absolute concentration and NAA/Cr ratio have been found in manic BD compared to healthy individuals in basal ganglia as well [109]. The NAA has been considered as a neuronal marker, and decreased NAA has been considered to indicate possible neuronal damage or loss [76]. Since NAA is also closely related to mitochondrial energy metabolism, the findings of reduced levels of NAA compared to HC could also be interpreted as indirect evidence for mitochondrial dysfunction in BD [106].

### 4.2. Polarity-Related Regional Specificity of Metabolite Changes within the Emotion Regulation Network

In our study, we found a preferential pattern of greater differences in PCC and HIPPO when OP-M and PP-M were compared to HC and in ACC when OP-D and PP-D were compared to HC. This pattern may imply a polarity-related regional specificity within the emotion regulation network, which needs to be further explored in future MRS studies.

The emotion regulation network involves several brain regions working together to effectively regulate emotions. Among these regions are the ACC, the PCC, and the HIPPO, with each of these regions playing a distinct yet interconnected role in emotion regulation [110]. The ACC has a crucial role in detecting emotional salience and initiating appropriate regulatory responses and is involved in emotion appraisal, conflict monitoring, and cognitive control. Subregions of the ACC may also have different functions in emotion regulation, with dorsal ACC being associated with cognitive control processes and rostral ACC with social and affective processing. The PCC is a key hub of the DMN. While traditionally related to internally directed processes, emerging evidence suggests that the PCC also contributes to emotion regulation by integrating self-relevant information with emotional experiences and facilitating adaptive responses to emotional stimuli. The HIPPO plays a crucial role in encoding, consolidating, and retrieving episodic memories, including emotionally salient events. It provides contextual information necessary for effective emotion regulation by linking current emotional experiences with past experiences stored in memory. Hippocampal dysfunction, such as structural alterations or impaired functioning, may disrupt the ability to regulate emotions in response to contextual cues and contribute to emotional dysregulation observed in BD. Interactions between these regions, along with other components of the emotion regulation network such as the prefrontal cortex and the amygdala, facilitate the flexible modulation of emotional responses in different contexts and are involved in the pathophysiology of BD [111]. Understanding the specific contributions of each region to emotion regulation can inform therapeutic interventions aimed at improving emotional well-being and resilience [112].

### 4.3. Metabolite Changes as Consistent Markers of Onset and Predominant Polarity Subphenotypes

Our follow-up analysis further revealed that the magnitude of OP-related differences (i.e., HC vs. OP-M, HC vs. OP-D, OP-M vs. OP-D) was considerably preserved in PP-related differences (i.e., HC vs. PP-M, HC vs. PP-D, PP-M vs. PP-D), since there was a high consistency of the effect sizes. OP is supposed to have a potential role in the BD course, outcome, prognosis, and impact on clinical and therapeutic decision-making. OP may contribute to identifying more homogeneous subgroups of BD patients [113], thus enabling the application of more targeted interventions [114]. OP has been linked to chronic severity of illness and treatment response [115,116], as well as other clinical variables, including rapid cycling [29], number of episodes and suicide attempts [28], comorbid psychiatric disorders [117], and lifetime psychotic symptoms [113]. Overall, patients with OP-D seem to have a worse prognosis [27].

However, to date, only limited evidence is available in relation to the neuroanatomical substrate of OP [47,48]. In a previous multimodal cerebellar study, our group found fractional anisotropy (FA) changes in (a) left/right contralateral fronto-ponto-cerebellar tracts (OP-D > HC) and (b) all fronto-ponto-cerebellar, most parieto-ponto-cerebellar and right contralateral occipito-ponto-cerebellar tracts (OP-M > HC) and generally observed greater and more widespread cerebro-cerebellar changes in OP-M patients than in OP-D patients compared to HC [47]. On the other hand, between-OP subgroup differences (OP-M > OP-D) were found in OP-M in several afferent WM tracts. Of note, regarding PP subgroups, we found FA changes in (a) left contralateral fronto-ponto-cerebellar tract (PP-D > HC) and (b) contralateral/ipsilateral fronto-ponto-cerebellar tracts bilaterally (PP-M > HC) [47]. In a recent whole-brain study, we found a main effect of OP on gray matter volume of the left middle frontal gyrus and of OP and PP (either or both) on the cortical thickness of various regions previously implicated in BD, i.e., inferior frontal gyrus-pars opercularis (left) and pars orbitalis (bilateral), left lateral orbitofrontal gyrus, a bilateral medial segment of the superior frontal gyrus, left planum polare, right anterior cingulate gyrus, left anterior and posterior insula, bilateral frontal operculum (both OP and PP); left anterior and posterior orbitofrontal gyrus, left transverse temporal gyrus, right posterior insula (only OP); and right medial frontal cortex (only PP) [48].

From a cognitive perspective, studies on cognition in BD suggest that cognitive deficits are already present in the early stages of BD, and some of them are state markers that change in association with BD status (e.g., relapses, euthymia, advanced stages) while others are genetic markers remaining relatively stable across the BD course [118]. The pattern of cognitive impairment in BD patients with different OP is less well-characterized. In a recent study, patients with OP-D showed the worst cognitive profile in measures of sustained attention, short-term and working memory, and cognitive flexibility [119]. These findings are in line with the pattern of differences observed in our study (Figure 3), highlighting greater differences in ACC metabolites in OP-D vs. HC compared to OP-M vs. HC.

In addition, even though OP has been associated with PP [28,29,120,121], no data are available regarding how the neuroanatomical substrate is related to OP and PP when both are studied together and whether any neuroanatomical changes can be considered as consistent markers of both OP and PP subphenotypes. In the absence of longitudinal data, one could speculate that our cross-sectional data reflect the consistency of polarity-related effects across the course of the disease, but this has to be tested in future longitudinal studies.

### 4.4. Study Limitations

Our study is not without limitations. First, the sample size of our study was relatively small, particularly in each subgroup of OP and especially PP. This may have decreased the power of comparison specifically when PP was considered. This also led us to rely on Cohen’s effect size for the identification of clinically significant differences. Further studies with larger sample sizes are necessary to reproduce/validate our results. Furthermore, this is a cross-sectional study, and we cannot substantiate a causal relationship between the observed changes and polarity subphenotypes. The patients in our study were not without medication, which could still cause a relative effect of medication on the metabolite profile in our sample [122]. A longitudinal study on euthymic patients considering their medication as a covariate of interest is warranted, since some drugs, such as lithium, are known to affect the levels of metabolite compounds in the brain [39,78,123]. However, it is worth noting that in our study, there were no differences in polarity subphenotypes with respect to lithium intake.

## 5. Conclusions

Our findings support the potential usefulness of MRS in the study of the neurobiological underpinnings of both OP and PP in BD, as significant differences and/or large effect sizes were observed between either HC and polarity subphenotypes or between manic and depressive polarity subphenotypes. We identified distributed patterns of metabolite–polarity associations that may differentiate polarity subphenotypes in BD and highlighted a potential regional specificity of manic and depressive polarity within the emotion regulation network. This pattern of changes consistently marked both OP and PP subphenotypes. The present results highlight the importance of studying both OP and PP in BD, expanding the existing field of neuroimaging studies on the neurobiological substrate of BD polarity and providing further evidence on the emotion dysregulation network in BD.

## Figures and Tables

**Table 1:** Demographic characteristics for all participants and clinical characteristics for patients with BD according to OP and PP subphenotypes.

	HC (*n* = 16)	OP-M (*n* = 17)	OP-D (*n* = 24)	PP-M (*n* = 12)	PP-D (*n* = 14)	PP-U (*n* = 15)
Demographic characteristics						
Age (yrs)	40.31 ± 10.05	42.53 ± 12.73	49.21 ± 10.91	38.00 ± 10.02	51.50 ± 11.13	48.47 ± 11.30
Sex (M/F)	6/10	9/8	11/13	5/7	7/7	8/7
Education (years)	14.44 ± 2.07	14.35 ± 3.66	14.42 ± 3.80	15.17 ± 2.86	14.64 ± 4.13	13.53 ± 3.93
Clinical characteristics						
BD-type (I/II)	-	17/0	13/11	12/0	7/7	11/4
Illness duration (years)	-	12.59 ± 8.93	16.25 ± 9.35	12.58 ± 8.11	15.71 ± 10.40	15.53 ± 9.30
Hospitalizations (*n*)	-	2.59 ± 2.00	2.33 ± 1.79	2.58 ± 1.98	2.43 ± 1.79	2.25 ± 1.92
Depressive episodes (*n*)	-	2.94 ± 4.84	4.50 ± 4.03	0.67 ± 0.78	4.21 ± 2.49	6.07 ± 5.91
Hyperthymis episodes (*n*)	-	4.47 ± 4.60	3.04 ± 2.96	3.50 ± 2.02	1.64 ± 0.84	5.60 ± 5.29
Lifetime suicidal attempts (Y/N)	-	2/15	15/9	4/8	7/7	6/9
Lifetime psychosis (Y/N)	-	12/5	10/14	9/3	4/10	9/6
Lifetime Axis I comorbidity (Y/N)	-	4/13	8/16	3/9	4/10	5/10
Current lithium (Y/N)	-	5/12	5/19	3/9	3/11	4/11
Current anticonvulsants (Y/N)	-	14/3	20/4	8/4	13/1	13/2
Current antidepressants (Y/N)	-	3/14	9/15	2/10	7/7	3/12
Current antipsychotics (Y/N)	-	15/2	20/4	10/2	12/2	13/2
FH-FDR of BD (Y/N)	-	5/12	3/21	2/10	4/10	2/13
FH-FDR of schizophrenia (Y/N)	-	2/15	1/23	1/11	1/13	1/14
FH-FDR of MDD (Y/N)	-	4/13	9/15	4/8	3/11	6/9

Notes. BD = bipolar disorder; HC = healthy controls; OP-M = manic onset polarity; OP-D = depressive onset polarity; PP-M = manic predominant polarity; PP-D = depressive predominant polarity; PP-U = unspecified predominant polarity; yrs = years; M/F = male/female; M = manic; D = depressive; U = unspecified; Y/N = yes/no; FH-FDR = family history in first-degree relatives; MDD = major depressive disorder.

**Table 2:** Crosstabulation of OP and PP subphenotypes.

	PP-M	PP-D	PP-U	Total
OP-M	9 (52.9%)	2 (11.8%)	6 (35.3%)	17
OP-D	3 (12.5%)	12 (50.0%)	9 (37.5%)	24
Total	12	14	15	41

Notes. Row percentages are presented. OP-M = manic onset polarity; OP-D = depressive onset polarity; PP-M = manic predominant polarity; PP-D = depressive predominant polarity; PP-U = unspecified predominant polarity.

**Table 3:** Profile of differences in ACC, PCC, LHIPPO, and RHIPPO in OP subgroups and HC.

Anatomical Region/Metabolite	E.M.M. ± S.E. for Groups			Statistics		
	HC	OP-M	OP-D	Univariate *p*-Value	Partial η^2^	Post Hoc Comparisons (Corrected *p* < 0.05)
Dependent variables: absolute values (tNAA, tCr, tCho, mI)				Pillai’s Trace Multivariate F = 1.672, *p* = 0.039		
ACC						
tNAA	8.44 ± 0.58	7.26 ± 0.54	8.53 ± 0.49			
tCr	10.58 ± 0.50	10.43 ± 0.46	11.23 ± 0.42			
tCho	2.89 ± 0.32	3.25 ± 0.30	3.43 ± 0.27			
mI	5.83 ± 0.58	6.02 ± 0.54	4.19 ± 0.49	**0.038**	0.128	(OP-M > OP-D, *p* = 0.056)
PCC						
tNAA	8.53 ± 0.80	7.83 ± 0.75	8.91 ± 0.68			
tCr	10.42 ± 0.41	11.67 ± 0.38	11.18 ± 0.34	(0.081)	0.099	(HC < OP-M, *p* = 0.078)
tCho	1.91 ± 0.39	3.26 ± 0.37	2.22 ± 0.33	**0.033**	0.133	HC < OP-M, *p* = 0.043
mI	5.39 ± 0.75	5.39 ± 0.70	4.67 ± 0.63			
LHIPPO						
tNAA	5.78 ± 0.58	5.49 ± 0.54	6.73 ± 0.49			
tCr	7.23 ± 0.47	6.85 ± 0.44	7.35 ± 0.40			
tCho	1.85 ± 0.26	2.13 ± 0.24	1.81 ± 0.22			
mI	8.69 ± 0.62	7.23 ± 0.58	9.11 ± 0.53	(0.061)	0.110	(OP-M < OP-D, *p* = 0.072)
RHIPPO						
tNAA	6.07 ± 0.52	4.92 ± 0.48	5.67 ± 0.44			
tCr	8.63 ± 0.63	6.56 ± 0.59	7.88 ± 0.53	(0.054)	0.115	(HC > OP-M, *p* = 0.056)
tCho	2.03 ± 0.23	2.52 ± 0.21	2.08 ± 0.20			
mI	9.90 ± 1.63	9.48 ± 1.52	11.00 ± 1.38			
Dependent variables: ratios (tNAA/tCr, tNAA/tCho, tCho/tCr, mI/tNAA, mI/tCr, mI/tCho)				Pillai’s Trace Multivariate F = 2.148, *p* = 0.004		
ACC						
tNAA/tCr	0.80 ± 0.05	0.72 ± 0.05	0.77 ± 0.04			
tNAA/tCho	3.06 ± 0.24	2.59 ± 0.22	2.70 ± 0.20			
tCho/tCr	0.27 ± 0.03	0.30 ± 0.02	0.31 ± 0.02			
mI/tNAA	0.71 ± 0.08	0.88 ± 0.08	0.51 ± 0.07	**0.003**	0.210	OP-M > OP-D, *p* = 0.002
mI/tCr	0.55 ± 0.06	0.62 ± 0.06	0.37 ± 0.05	**0.005**	0.195	OP-M > OP-D, *p* = 0.005; (HC > OP-D, *p* = 0.076)
mI/tCho	2.21 ± 0.24	2.22 ± 0.23	1.25 ± 0.21	**0.004**	0.203	HC > OP-D, *p* = 0.016; OP-M > OP-D, *p* = 0.010
PCC						
tNAA/tCr	0.80 ± 0.06	0.67 ± 0.06	0.80 ± 0.05			
tNAA/tCho	4.54 ± 0.45	3.89 ± 0.42	4.12 ± 0.38			
tCho/tCr	0.19 ± 0.04	0.28 ± 0.03	0.20 ± 0.03			
mI/tNAA	0.80 ± 0.29	1.06 ± 0.27	0.71 ± 0.24			
mI/tCr	0.53 ± 0.08	0.45 ± 0.07	0.44 ± 0.07			
mI/tCho	2.84 ± 0.35	2.27 ± 0.33	2.14 ± 0.30			
LHIPPO						
tNAA/tCr	0.84 ± 0.08	0.81 ± 0.08	0.94 ± 0.07			
tNAA/tCho	3.39 ± 0.33	3.18 ± 0.31	3.77 ± 0.28			
tCho/tCr	0.25 ± 0.05	0.35 ± 0.05	0.25 ± 0.04			
mI/tNAA	1.93 ± 0.31	1.49 ± 0.29	1.60 ± 0.26			
mI/tCr	1.29 ± 0.13	1.08 ± 0.12	1.31 ± 0.11			
mI/tCho	5.69 ± 0.56	4.28 ± 0.52	5.18 ± 0.47			
RHIPPO						
tNAA/tCr	0.76 ± 0.09	0.85 ± 0.09	0.76 ± 0.08			
tNAA/tCho	3.18 ± 0.26	2.28 ± 0.24	2.90 ± 0.22	**0.035**	0.130	HC > OP-M, *p* = 0.038
tCho/tCr	0.25 ± 0.03	0.37 ± 0.03	0.27 ± 0.02	**0.005**	0.201	HC < OP-M, *p* = 0.008; OP-M > OP-D, *p* = 0.024
mI/tNAA	1.73 ± 0.69	2.12 ± 0.64	2.37 ± 0.58			
mI/tCr	1.28 ± 0.24	1.66 ± 0.23	1.38 ± 0.21			
mI/tCho	5.26 ± 0.59	4.12 ± 0.55	5.21 ± 0.50			

Notes. ACC = anterior cingulate cortex; PCC = posterior cingulate cortex; LHIPPO = left hippocampus; RHIPPO = right hippocampus; OP = onset polarity; OP-M = manic onset polarity; OP-D = depressive onset polarity; HC = healthy controls; E.M.M. = estimated marginal means; S.E. = standard error; tNAA = total NAA [N-acetylaspartate (NAA) + N-acetylaspartateglutame (NAAG)]; tCr = total Creatine [creatine (CR) + Phospho-creatine (PCR)]; tCho = total Choline [Glycerophosphocholine (GPC) + Phosphocholine (PCH)]; mI = myo-Inositol. E.M.M. ± S.E. for spectroscopic values are adjusted for age, sex, and education. In the case of a significant MANCOVA Pillai’s Trace, metabolites (absolute values or ratios) with a significant univariate omnibus test (main effect) were identified, and post hoc comparisons between subgroups were performed applying Bonferroni correction for multiple tests. Bold *p*-values are significant at *p* < 0.05, following Bonferroni correction while *p*-values into brackets correspond to marginally significant univariate *p*-values (main effect) or post hoc comparisons *p*-values after Bonferroni correction (marginally significant *p*-value: 0.05–0.1). Partial η^2^ effect size is interpreted as small (η^2^p = 0.01), medium (η^2^p = 0.06), or large (η^2^p = 0.14).

**Table 4:** Profile of differences in ACC, PCC, LHIPPO, and RHIPPO in PP subgroups and HC.

Anatomical Region/Metabolite	E.M.M. ± S.E. for Groups				Statistics		
	HC	PP-Μ	PP-D	PP-U	Univariate *p*-Value	Partial η^2^	Post hoc Comparisons
Dependent variables: absolute values (tNAA, tCr, tCho, mI)					Pillai’s Trace Multivariate F = 1.052, *p* = 0.408		
ACC							
tNAA	8.37 ± 0.56	6.58 ± 0.65	8.22 ± 0.60	9.09 ± 0.61			
tCr	10.57 ± 0.51	10.41 ± 0.58	11.18 ± 0.54	11.01 ± 0.55			
tCho	2.91 ± 0.31	3.57 ± 0.36	3.60 ± 0.33	2.85 ± 0.34			
mI	5.76 ± 0.61	5.13 ± 0.70	4.29 ± 0.64	5.69 ± 0.66			
PCC							
tNAA	8.52 ± 0.81	7.88 ± 0.94	9.24 ± 0.86	8.11 ± 0.88			
tCr	10.37 ± 0.41	11.17 ± 0.48	11.39 ± 0.44	11.65 ± 0.45			
tCho	1.86 ± 0.40	2.60 ± 0.46	2.26 ± 0.43	3.24 ± 0.44			
mI	5.32 ± 0.76	4.69 ± 0.88	5.03 ± 0.81	5.27 ± 0.83			
LHIPPO							
tNAA	5.85 ± 0.60	6.23 ± 0.69	6.36 ± 0.64	5.90 ± 0.65			
tCr	7.22 ± 0.48	6.80 ± 0.55	7.48 ± 0.51	7.09 ± 0.52			
tCho	1.86 ± 0.26	2.25 ± 0.30	1.78 ± 0.28	1.84 ± 0.28			
mI	8.72 ± 0.45	7.62 ± 0.76	8.56 ± 0.70	8.58 ± 0.71			
RHIPPO							
tNAA	6.06 ± 0.53	4.90 ± 0.61	5.57 ± 0.56	5.53 ± 0.57			
tCr	8.56 ± 0.62	6.00 ± 0.71	8.44 ± 0.66	7.37 ± 0.67			
tCho	2.01 ± 0.24	2.26 ± 0.27	2.46 ± 0.25	2.10 ± 0.26			
mI	9.75 ± 1.63	8.02 ± 1.88	11.32 ± 1.73	11.60 ± 1.76			
Dependent variables: ratios (tNAA/tCr, tNAA/tCho, tCho/tCr, mI/tNAA, mI/tCr, mI/tCho)					Pillai’s Trace Multivariate F = 1.218, *p* = 0.196		
ACC							
tNAA/tCr	0.79 ± 0.05	0.66 ± 0.06	0.74 ± 0.05	0.84 ± 0.05			
tNAA/tCho	3.03 ± 0.22	2.20 ± 0.26	2.51 ± 0.24	3.25 ± 0.24			
tCho/tCr	0.28 ± 0.02	0.33 ± 0.03	0.33 ± 0.03	0.26 ± 0.03			
mI/tNAA	0.70 ± 0.09	0.83 ± 0.10	0.57 ± 0.09	0.64 ± 0.10			
mI/tCr	0.55 ± 0.07	0.53 ± 0.07	0.38 ± 0.07	0.55 ± 0.07			
mI/tCho	2.18 ± 0.26	1.71 ± 0.30	1.30 ± 0.28	2.08 ± 0.28			
PCC							
tNAA/tCr	0.81 ± 0.06	0.71 ± 0.07	0.81 ± 0.07	0.69 ± 0.07			
tNAA/tCho	4.54 ± 0.45	4.04 ± 0.52	4.42 ± 0.48	3.57 ± 0.49			
tCho/tCr	0.18 ± 0.04	0.23 ± 0.04	0.20 ± 0.04	0.28 ± 0.04			
mI/tNAA	0.76 ± 0.29	0.61 ± 0.33	0.90 ± 0.31	1.11 ± 0.31			
mI/tCr	0.53 ± 0.08	0.42 ± 0.09	0.48 ± 0.09	0.44 ± 0.09			
mI/tCho	2.85 ± 0.36	2.35 ± 0.41	2.28 ± 0.38	1.97 ± 0.39			
LHIPPO							
tNAA/tCr	0.84 ± 0.09	0.91 ± 0.10	0.87 ± 0.09	0.86 ± 0.09			
tNAA/tCho	3.43 ± 0.34	3.61 ± 0.39	3.52 ± 0.36	3.38 ± 0.37			
tCho/tCr	0.26 ± 0.06	0.38 ± 0.06	0.24 ± 0.06	0.26 ± 0.06			
mI/tNAA	1.91 ± 0.31	1.23 ± 0.36	1.66 ± 0.33	1.77 ± 0.33			
mI/tCr	1.29 ± 0.13	1.18 ± 0.15	1.22 ± 0.14	1.23 ± 0.15			
mI/tCho	5.71 ± 0.57	4.56 ± 0.66	4.81 ± 0.61	4.96 ± 0.62			
RHIPPO							
tNAA/tCr	0.77 ± 0.09	0.93 ± 0.11	0.72 ± 0.10	0.75 ± 0.10			
tNAA/tCho	3.21 ± 0.27	2.54 ± 0.31	2.52 ± 0.29	2.79 ± 0.29			
tCho/tCr	0.25 ± 0.03	0.36 ± 0.04	0.30 ± 0.03	0.28 ± 0.03			
mI/tNAA	1.68 ± 0.69	1.63 ± 0.79	2.70 ± 0.73	2.43 ± 0.75			
mI/tCr	1.28 ± 0.25	1.66 ± 0.28	1.32 ± 0.26	1.55 ± 0.27			
mI/tCho	5.27 ± 0.61	4.26 ± 0.70	4.54 ± 0.64	5.36 ± 0.66			

Notes. ACC = anterior cingulate cortex; PCC = posterior cingulate cortex; LHIPPO = left hippocampus; RHIPPO = right hippocampus; PP = predominant polarity; PP-M = manic predominant polarity; PP-D = depressive predominant polarity; PP-U = unspecified predominant polarity; HC = healthy controls; E.M.M. = estimated marginal means; S.E. = standard error; tNAA = total NAA [N-acetylaspartate (NAA) + N-acetylaspartateglutame (NAAG)]; tCr = total Creatine [creatine (CR) + Phospho-creatine (PCR)]; tCho = total Choline [Glycerophosphocholine (GPC) + Phosphocholine (PCH)]; mI = myo-Inositol. E.M.M. ± S.E. for spectroscopic values are adjusted for age, sex, and education. Post hoc univariate comparisons between groups were not performed because the MANCOVA Pillai’s Trace multivariate test was not significant.

**Table 5:** Magnitude of differences (Cohen’s d effect sizes) for ACC, PCC, LHIPPO, and RHIPPO in OP and PP between-group comparisons.

	HC vs. OP-M	HC vs. OP-D	OP-M vs. OP-D	HC vs. PP-M	HC vs. PP-D	HC vs. PP-U	PP-M vs. PP-D	PP-M vs. PP-U	PP-D vs. PP-U
ACC									
mI	−0.08	0.72	0.81	0.26	0.62	0.03	0.35	−0.23	−0.59
mI/tNAA	−0.53	0.63	1.12 *	−0.37	0.38	0.17	0.76	0.54	−0.20
mI/tCho	−0.29	0.77	1.04 *	0.08	0.64	0.00	0.59	−0.08	−0.66
mI/tCr	−0.01	1.00 *	1.00 *	0.46	0.86	0.10	0.39	−0.36	−0.76
PCC									
tCr	−0.79	−0.48	0.31	−0.49	−0.63	−0.80	−0.13	−0.29	−0.16
tCho	−0.89 *	−0.20	0.68	−0.47	−0.25	−0.88	0.21	−0.40	−0.61
LHIPPO									
mI	0.61	−0.17	−0.77	0.49	0.07	0.06	−0.36	−0.37	−0.01
RHIPPO									
tCr	0.85	0.30	−0.54	1.05	0.05	0.49	−0.99	−0.56	0.44
tNAA/tCho	0.90 *	0.27	−0.61	0.63	0.65	0.40	0.02	−0.24	−0.25
tCho/tCr	−1.00 *	−0.19	0.91 *	−0.86	−0.44	−0.27	0.48	0.64	0.18

Notes. HC = healthy controls; OP-M = manic onset polarity; OP-D = depressive onset polarity; PP-M = manic predominant polarity; PP-D = depressive predominant polarity; PP-U = unspecified predominant polarity; ACC = anterior cingulate; PCC = posterior cingulate; LHIPPO = left hippocampus; RHIPPO = right hippocampus; mI = myo-Inositol; tNAA = total NAA [N-acetylaspartate (NAA) + N-acetylaspartateglutame (NAAG)]; tCr = total Creatine [creatine (CR) + Phospho-creatine (PCR)]; tCho = total Choline [Glycerophosphocholine (GPC) + Phosphocholine (PCH)]. * Effect sizes that were accompanied by significant *p*-values after correction for multiple comparisons in previous post hoc analyses. Cells with brown color represent large effect sizes (|d| ≥ 0.80), cells with orange color represent medium effect sizes (0.79 ≤ |d| ≥ 0.50) while non-colored cells represent small (0.49 ≥ |d| ≥ 0.20) or negligible (|d| ≤ 0.19) effect sizes.

## Data Availability

Data are available upon request.
